# Attitudes and barriers associated with seasonal influenza vaccination uptake among public health students; a cross-sectional study

**DOI:** 10.1186/s12889-018-6041-1

**Published:** 2018-09-20

**Authors:** Christopher J. Rogers, Kaitlin O. Bahr, Stephanie M. Benjamin

**Affiliations:** Department of Health Sciences, California State University, Northridge, 18111 Nordhoff St., Northridge, CA 91330 USA

**Keywords:** Seasonal influenza vaccine, Public health, College, Education, Vaccine misperceptions

## Abstract

**Background:**

Although research has explored influenza vaccination uptake among medical and college students, there is a dearth of research in understanding influenza vaccination uptake and attitudes toward the vaccine among future public health practitioners. Undergraduate public health students represent future public health practitioners who may be a significant educational resource for health information, including the importance of vaccinations.

**Methods:**

This cross-sectional study utilized survey data from 158 undergraduate public health students attending a large public university in Southern California. The survey assessed public health students’ attitudes and beliefs towards the seasonal influenza vaccine and seasonal vaccination rates among this population.

**Results:**

Over 88% of respondents reported having been encouraged to receive the seasonal influenza vaccine, while only 43.0% reported receipt. Of the students who reported not receiving the vaccine, 49.4% believed it may give them the flu, 30.4% believed there may be dangerous side effects, and 28.9% believed they were not at risk for contracting the flu. Access to health care practitioners (OR: 3.947, 95% CI [1.308–11.906]) and social encouragement (OR: 3.139, 95% CI [1.447–6.811]) were significantly associated with receipt of the seasonal influenza vaccine.

**Conclusion:**

As public health program curriculum includes information about seasonal influenza vaccination and 68% of the sample were seniors soon to be exiting the program with an undergraduate degree in public health education, this low seasonal influenza vaccination rate is disturbing. This study may add to the body of data demonstrating how knowledge of the vaccine does not always guarantee vaccine uptake. Results of the current study suggest that it may be beneficial to provide additional information targeted to public health students, aimed at mediating safety concerns and increasing social pressure to assist in improving vaccine acceptance and rates in this population. Maximizing seasonal influenza vaccination uptake by addressing attitudes, barriers and misperceptions may not only improve vaccination rates among public health students, but also in communities served by these future public health practitioners.

## Background

Influenza is a potentially deadly virus with a seasonal peak between late fall and early spring [1]. In the United States (US), there are approximately 55,000 to 431,000 cases of influenza-associated hospitalizations per year [1]. The Centers for Disease Control and Prevention (CDC) recommends routine annual seasonal influenza vaccinations for all persons 6 months and older with vaccination of particular importance to vulnerable populations, including older adults, young children, pregnant women, and persons with chronic medical conditions, for whom influenza can cause serious illness or death [1, 2]. Despite these recommendations, US seasonal influenza vaccination rates remain low. It is estimated that only 41% of adults receive the vaccine, an increase of only 3% in the past 7 years, falling short of the US Healthy People 2020 goal of 70% [3, 4].

Health care workers represent an especially vulnerable population when it comes to risk of exposure to the influenza virus. To limit the risk of influenza in this high-risk population, Healthy People 2020 set a goal of 90% for health care worker vaccination rates, which were estimated to range from 64.3 to 75.2% across the 2013–14 full season, and the 2014–15 early season [5–7]. Misconceptions about the vaccine have contributed to low influenza vaccination rates in the US, including among health care workers [5, 7]. Doubting the effectiveness of the vaccine and fear of the vaccine causing illness were the most common reasons given for avoiding vaccination [5, 7]. Physicians, health educators, and other health practitioners are integral to raising community awareness, improving vaccination education, and developing campaigns to increase vaccination uptake among the general population and other health care workers [8–11]. Improving our understanding of the attitudes regarding vaccination as well as factors related to vaccine uptake among health care workers and public health practitioners would be useful in creating more effective educational materials and plans for distribution that may dispel myths about the vaccine within this population. To achieve the goal of improving influenza vaccination rates, it is critical for public health practitioners, including health educators, to be well informed of the benefits and importance of the influenza vaccine.

Several studies have examined seasonal influenza vaccination rates specifically among medical students and residents. US medical student and resident seasonal influenza vaccination rates have been found to range from 48 to 58% [12–17]. Outside of studies focused on students pursuing medical degrees, there have been few assessing influenza vaccination habits of general college students, with existing research finding vaccination rates from 8 to 30% [18–24]. Continued research is needed in college students, as influenza has the potential to threaten student populations due to ease of transmission through close social contact and the close proximity of living conditions [25–29]. Although research has explored influenza vaccination uptake among medical students, college students, and health care workers, there is a dearth of research in understanding influenza vaccination uptake and attitudes toward the vaccine among future public health practitioners and future health care workers. Undergraduate public health students represent an important part of the future public health workforce who may be a significant educational resource for health information, including the importance of vaccinations. It is essential to assess vaccination coverage, attitudes, and beliefs among this specific population who may in the future be working with or educating susceptible populations. In addressing the gap, this study assesses undergraduate public health student vaccination rates, attitudes and beliefs about the vaccine and factors impacting vaccination uptake.

## Methods

### Participants, study design, and aim

Participants were undergraduate students recruited from California State University, Northridge (CSUN), a large diverse public university in Southern California serving 41,548 students [30]. Undergraduates in 2015 were 54% female, 44% Hispanic, 53% low income students, and had a mean age of 23 [30]. This cross-sectional study was implemented in May 2015 using an anonymized self-completed electronic questionnaire. Following institutional review board approval, the questionnaire was emailed to undergraduate students majoring in public health. Eligible participants had to be at least 18 years of age and able to read and write in English. A total of 161 questionnaires were completed and 158 were included in analysis based on the inclusion criteria. Without any incentive or additional encouragement, the response rate was approximately 40%. The current study sought to explore three specific aims.

**Aim 1:** Assess the prevalence of seasonal influenza vaccination uptake and analyze potential differences in subgroups.

**Aim 2:** Explore potential barriers to receipt of the seasonal influenza vaccine.

**Aim 3:** Identify factors associated with reported receipt of the seasonal influenza vaccination.

### Measures and instrumentation

The questionnaire was adapted from a previous 2014 study assessing seasonal influenza vaccination coverage, as well as vaccine attitudes and barriers among undergraduate students at CSUN [24]. The questionnaire was redesigned to be in electronic format and administered through an online survey response collection program. These data were then downloaded and recorded for analysis.

Respondents completed a 26-item questionnaire which collected demographic information (including age, sex, race/ethnicity, on/off campus living situation, year of study, and questions relating to inclusion criteria) as well as vaccination history, beliefs about vaccinations, access to health insurance, and sources of vaccine information and vaccine uptake encouragement. Access to health insurance was assessed through three questions related to the use of the campus student health center, last medical check-up, and if the respondent had health insurance. Information on vaccination history was collected by three questions assessing lifetime receipt of influenza vaccination, past 10-month receipt of influenza vaccination, and location of past 10-month vaccination receipt. Questions related to information received about the vaccine and encouragement to get the influenza vaccine were adapted from a previous study and tailored to fit the studied institution [22]. The sources of encouragement and information included medical practitioners, the student health center, campus flyers/posters, off campus media, and relatives/friends.

Respondents who reported not receiving a vaccination within the past 10 months were asked to complete questions regarding their attitudes toward seasonal influenza vaccination. A Likert scale from 1 = strongly disagree to 4 = strongly agree was used to measure attitudes about the seasonal influenza vaccine. The scale was applied to assess the respondent’s level of agreement with statements relating to beliefs about vaccination cost, adverse events, importance, risk, and religious or cultural barriers. Questions on attitudes about the vaccine were originally adapted from a previous study conducted on a college campus to examine factors and barriers associated with receipt of the influenza vaccine [22].

### Analyses

Frequency distributions and descriptive statistics were used to assess demographic variables. Associations between demographic factors and vaccination coverage were calculated using t-tests (age) and chi-square tests (race/ethnicity, campus residence, and year of undergraduate study). Statements about beliefs relating to cost, access, and risks of vaccination were truncated to percentage of agreement (either “Agree” or “Strongly Agree”) and percentage of disagreement (either “Disagree” or “Strongly Disagree”). The statements about attitudes and beliefs were ranked according to percentage of agreement, and full response means and standard deviations were calculated from all four Likert scale response options. To assess the relationship of factors influencing influenza vaccination and the receipt of the current seasonal influenza vaccine, a logistic regression model was used. Model covariates were selected a priori based on previous study [22, 24]. Demographic covariates were limited due to the small sample size. The multivariable model, adjusting for age, included time elapsed since the most recent visit to a medical provider and sources of vaccine encouragement and information; all of which were regressed on past 10-month influenza vaccine uptake. Analyses were completed using SPSS software version 24, with a statistical significance level set to 0.05 [31].

## Results

Respondents were 158 undergraduate public health students aged 18 to 54 with a mean age of 22.49 (SD = 3.674); 90.5% identified as female, and the most frequently reported race/ethnicity was Hispanic or Latino (50.3%), followed by Asian or Pacific Islander (16.6%). Most of the respondents reported that they were seniors within the public health program (67.7%) and 96.2% reported living off campus.

Almost half (48.4%) of all respondents reported use of the campus student health center within the past 10 months. Over 58% of respondents reported having seen a medical provider within the past 6 months and 86.6% reported having some form of health insurance. The majority (88.4%) of the respondents reported having been encouraged to receive the seasonal influenza vaccine. The overall reported receipt of the seasonal influenza vaccine was 43.0% of respondents. Demographic variables stratified by reported receipt of the seasonal influenza vaccine are presented in Table 1. A significant association was found between receipt of the vaccine and when respondents had last visited a medical provider (*p* < 0.001). Age, sex, race/ethnicity, residence, year of undergraduate study, and access to health insurance were not found to be significantly associated with receiving the vaccine.

**Table 1:** Demographic and health related characteristics of respondents by receipt of seasonal influenza vaccine (within the past 10 months)

	Received the vaccine 푛 (%)	Did not receive the vaccine 푛 (%)	푝 value*
Total	64 (43.0)	84 (57.0)	–
Age mean (SD)	22.69 (3.039)	22.10 (2.397)	0.189
Sex			
Male	5 (35.7)	9 (64.3)	0.381
Female	59 (44.0)	75 (56.0)	
Race/ethnicity			
White/Caucasian	7 (38.9)	11 (61.1)	0.199
Black/African American	1 (10.0)	9 (90.0)	
Hispanic/Latino	38 (49.4)	39 (50.6)	
Asian/Pacific Islander	11 (45.8)	13 (54.2)	
Multiracial/Other	7 (38.9)	11 (61.1)	
Campus residence			
On campus	3 (60.0)	2 (40.0)	0.366
Off campus	60 (42.3)	82 (57.7)	
Year of undergraduate study			
Freshman / Sophomore	10 (52.6)	9 (47.4)	0.292
Junior	8 (30.8)	18 (69.2)	
Senior	46 (45.1)	56 (54.9)	
Health insurance			
Yes, I have health insurance	52 (40.9)	75 (59.1)	0.174
No, I do not have health insurance	11 (55.0)	9 (45.0)	
The last time you went to a medical provider for treatment or a check-up			
In the past month	18 (54.5)	15 (45.5)	< 0.001
In the past 6 months	33 (61.1)	21 (38.9)	
Within the past 6 to 12 months	8 (27.6)	21 (72.4)	
More than 1 year / I do not remember the last time	5 (15.6)	27 (84.4)	

*푝 values were calculated using 푡-tests (age) and 휒^2^ tests (sex, race/ethnicity, campus residence, year of undergraduate study, health insurance, and the last time you went to a medical provider for treatment or a check-up.), with a significance level of 0.05

Statements regarding barriers to receipt of the seasonal influenza vaccine, ranked by percent of agreement, are presented in Table 2. Among students who reported not receiving a seasonal influenza vaccination within the last 10 months: 49.4% of students agreed with the statement “I believe that as a result of the flu shot I may actually get the flu,” 44.9% of students agreed with the statement “I do not have time to get a flu vaccination,” 30.4% of students agreed with the statement “I believe that vaccines may have dangerous side effects,” 28.9% of students agreed with the statement “I do not believe I am in danger of contracting the flu,” 10.7% of students agreed with the statement “I was not informed that flu vaccines might be important,” 9.5% of students agreed with the statement “I do not know where to receive a flu vaccination,” 9.1% of students agreed with the statement “I do not believe in vaccines for religious or cultural reasons,” and 7.7% of students agreed with the statement “Vaccines are too expensive for me right now.” Percent of agreement did not significantly differ across gender, ethnicity, age, or year of undergraduate study.

**Table 2:** Potential barriers^a^ to receipt of the seasonal influenza vaccine

	n	Mean (sd)	% Agreement	Rank^b^
Cost				
Vaccines are too expensive for me right now	78	1.58 (0.635)	7.7	8
Access				
I do not have time to get a flu vaccination	78	2.27 (0.963)	44.9	2
I do not know where to receive a flu vaccination	74	1.57 (0.704)	9.5	6
Safety				
I believe that as a result of the flu shot I may actually get the flu	79	2.46 (1.023)	49.4	1
I believe that vaccines may have dangerous side effects	79	2.05 (0.959)	30.4	3
Perceived Importance				
I was not informed that flu vaccines might be important	75	1.67 (0.741)	10.7	5
I do not believe I am in danger of contracting the flu	76	2.12 (0.879)	28.9	4
Other				
I do not believe in vaccines for religious or cultural reasons	77	1.52 (0.736)	9.1	7

^a^Barriers were assessed on a Likert scale of agreement from 1 (Strongly Disagree) to 4 (Strongly Agree) and ranked based on percentages of statement agreement (scores of 3–4)

^b^Ranked in order of percent of agreement with the referenced statements

Note: This table represents responses of students who reported they did not receive the vaccine

Multivariate regression analysis identifying factors associated with receipt of seasonal influenza vaccinations is presented in Table 3. Two factors were identified as significantly associated with receipt of seasonal influenza vaccinations: seeing a medical provider within the last 6 months (OR: 3.947, 95% CI [1.308–11.906], *p* = 0.015) and being encouraged by parents, relatives, or friends (OR: 3.139, 95% CI [1.447–6.811], *p* = 0.004).

**Table 3:** Multivariate analysis of factors associated with reported receipt of the seasonal influenza vaccination (within 10 months preceding data collection)

	Odds ratio (95% confidence interval)
Encouraged by parents, relatives, or friends	3.139** (1.447–6.811)
Saw a medical provider within the last 6 months	3.947* (1.308–11.906)
Encouraged by flyers, TV, or billboards	1.188 (.360–3.924)
Encouraged by a medical professional	1.087 (.379–3.114)
Age	1.108 (.954–1.285)

*푝< 0.05, **푝< 0.01, ***푝< 0.001

Model controls for age and other covariates

## Discussion

The current study explored the seasonal influenza vaccination rate, attitudes and beliefs toward the seasonal influenza vaccine, and factors associated with vaccine uptake among undergraduate students majoring in public health. Results demonstrated that less than half of students surveyed (43%) reported receipt of the seasonal influenza vaccine. The findings are lower than many of the previous studies which focused on medical students [12–17]. These low uptake results were concerning, particularly considering 68% of the sample were seniors soon to be exiting the program with an undergraduate degree in public health education. Even though the core curriculum of the public health program covered topics such as seasonal influenza vaccination, studies have demonstrated that knowledge of behaviors do not always guarantee behavior change [22, 32–34]. A 2014 study, at the same university, investigating seasonal influenza vaccination coverage and attitudes and beliefs toward the vaccine among undergraduate students of all majors, found only 20.6% of students reported receiving the vaccine and nearly 50% had unsubstantiated fears about vaccination (Benjamin & Bahr, 2016).

Age was not found to be significantly associated with reported receipt of the seasonal influenza vaccine in the current study. However, a significant association was found between reported receipt of the seasonal influenza vaccine and “The last time you went to a medical provider for treatment or a check-up” *p* = 0.004, OR: 3.947, 95% CI [1.308–11.906]). There was a significantly greater proportion of students reporting receipt of the seasonal influenza vaccine among respondents reporting seeing a medical provider within the past 6 months, compared to those who did not see a medical provider within the past 6 months, 58.6% to 21.3% respectively (*p* < 0.001). An explanation could be that students who have seen a medical provider within the past 6 months may be more likely to have health insurance, access to medical services, or are more likely to be encouraged to receive the vaccine by their medical provider. Among those reporting having not received encouragement from parents, relatives, or friends, 73.8% did not receive the seasonal influenza vaccine. Previous studies in college populations also identified receiving encouragement or information from a medical professional to be associated with vaccination uptake [22, 24]. However, encouragement or information from a medical professional was not found to be significant in multivariate modeling. This could be explained by the fact that the student’s curriculum typically covered vaccination recommendations from medical professionals. Since this is already a part of the core curriculum, the weight of influence from receiving encouragement or information from a personal physician or nurse may shift toward other sources. Instead, the model identified receiving encouragement from social groups (*p* = 0.015, OR = 3.139, 95% CI [1.447–6.811]) to be significant. These findings, confirmed by previous literature, indicate that interventions in this population that consider elements of social influence as well as access to health care, might have a significant impact on vaccination rates [22, 24, 35].

Among students who reported not receiving the seasonal influenza vaccination, a substantial proportion report safety as a concern. Nearly half of all respondents report a belief that the flu shot may give them the flu, with a third of respondents reporting the belief that there may be dangerous side effects, as well as a third reporting the belief that they are not even at risk for contracting the flu. This is in direct contrast to the information provided to students in the classroom during their time in the program and information provided by the CDC. The CDC clearly identified that an individual is not at risk of contracting influenza from the injected inactivated influenza vaccine and that there is a very remote chance of serious side effects in most populations [36]. Even with the abundance of information available in and out of the classroom, respondents had misconceptions about the safety of vaccinations. The results of the current study suggest that it may be beneficial to provide additional information targeted to public health students, with the goal of mediating safety concerns and potentially increasing vaccination rates in this population. These dialogues could take place in the classroom as a forum discussing the risks of both influenza as well as vaccination. This communication strategy could increase the social pressure to vaccinate while addressing safety concerns. The least frequently reported barriers were cost, not knowing where to get a vaccination, and religious or cultural objections. This suggests that within this population, cost and culture were not prohibitive factors and that students have an adequate knowledge of where to affordably access the influenza vaccinations; therefore, it may be beneficial to focus on providing these students with additional information about why the vaccine is important and safe, in addition to further information on where to receive it.

### Limitations

Study limitations include the use of a convenience sample administered through a department listserv. There is the possibility that students who participated were different from those who did not. Students that participated in the study were likely to be more interested in vaccination, which may have led to an overestimate of vaccination coverage and knowledge than present in non-participants. It is important to note that even with this possible overestimate of coverage, past-year seasonal vaccination coverage rates of 43.0% in the study population was still substantially lower than the CDC recommendations of routine annual seasonal influenza vaccinations for all persons 6 months and older. Additionally, without any incentive or additional encouragement, the response rate was approximately 40%, and those responding were predominantly female (90.5%), Hispanic (50.3%) and seniors within the public health program (67.7%). The distribution of gender and race/ethnicity is representative of the gender distribution and race/ethnicity distribution among students majoring in public health for the same year, (58.1% Hispanic/Latino and 82.7% female) [30]. More study participants were seniors (67.7%) compared with the approximately the approximately 22.9% of students that were seniors in the public health program during that same year [30]. Using self-reported data is another potential limitation and reporting bias may have been introduced into this study. Since students could have possibly felt pressured to respond positively to questions regarding vaccination uptake, an effort was made to mitigate overestimation of positive responses by making the questionnaire anonymous and not including sensitive issues. Finally, the issue of influenza vaccine effectiveness and how it may influence the decision making process of potential vaccination recipients was not addressed. It is important to consider that vaccine effectiveness may play a role in the decision making process of those who choose not to vaccinate and may be more pronounced among populations informed about vaccine effectiveness, such as health care workers. Further research is needed to explore if knowledge of vaccine effectiveness impacts vaccination uptake to establish a more comprehensive understanding of why some choose not to vaccinate.

Future studies within this population should consider investigation into whether other factors, such as sex, race, or income, could play a role in the vaccination coverage outcomes. Due to the sample size and low number of students who received the vaccine, the multivariable model was not sufficiently powered to handle additional demographic or other covariates. Because of this, only age and the variables included in the research hypothesis were modeled, since age had been a factor associated in previous literature [24]. Any conclusions drawn from this study must also be considered in light of the small sample size. Further research is needed within this population to determine if these relationships hold in a larger, more generalizable sample.

## Conclusion

Although public health students should be receiving information about vaccinations within their curriculum, it cannot be assumed that they will have positive attitudes and beliefs toward the seasonal influenza vaccine. Additionally, study has shown that knowledge alone may not be enough to increase uptake; attitudes about the value and risk, as well as misperceptions, may play a significant role [7, 37–39]. These study findings support the need for targeted intervention within college public health programs. Interventions that consider elements of social influence as well as access to health care might have an impact on vaccination rates in this population. Specific efforts to educate public health students and address the risks and importance of seasonal influenza vaccine uptake may assist in correcting misinformation. Influenza vaccine education strategies that encourage receipt, while incorporating medically accurate information from a nurse or physician, could be integral to correcting attitudinal misperceptions and increasing seasonal influenza vaccination coverage within public health students. Additional information included in curriculum should focus on changing the perceived risk and impact of acquiring seasonal influenza as well as addressing the limited risks from receipt of the vaccination. Many public health students will move into careers in health and medicine and may be the professionals depended upon to promote the vaccine. Maximizing seasonal influenza vaccination uptake in this population by addressing attitudes, barriers, and misperceptions may not only help improve vaccination rates in public health students now, but also in the communities served by these prospective health practitioners.

## Abbreviations

CDC: Centers for Disease Control and Prevention
CSUN: California State University, Northridge
OR: Odds Ratio
SD: Standard Deviation
US: United States
